# Age and diet affect self-resilience of intestinal microbiome in mice

**DOI:** 10.3389/fmicb.2025.1507396

**Published:** 2025-01-29

**Authors:** Chenyi Shao, Shenmin Chen, Huan Yang, Mufan Li, Yinhui Liu, Shu Wen, Jing Xiao, Li Tang

**Affiliations:** ^1^Department of Microecology, College of Basic Medical Sciences, Dalian Medical University, Dalian, China; ^2^Clinical Laboratory, Tianjin Children’s Hospital, Tianjin, China; ^3^Advanced Institute for Medical Sciences, Dalian Medical University, Dalian, China; ^4^Department of Oral Pathology, College of Stomatology, Dalian Medical University, Dalian, China

**Keywords:** gut microbiota, antibiotic-induced dysbiosis, self-resilience, age, lifespan, dietary

## Abstract

**Background:**

Gut microbiota contributes to human health. Little is known about the self-resilience of the gut microbiota after dysbiosis. This study aimed to investigate the self-resilience of the gut microbiome at different ages and the effects of diet on its recovery capacity in adulthood.

**Methods:**

A rodent model of antibiotic-induced dysbiosis was used. Microscopy was used to observe morphological changes in the mucosa. In addition, 16S rRNA sequencing and polymerase chain reaction-denaturing gradient gel electrophoresis were performed to identify the bacterial taxa and microbiome structure, respectively.

**Results:**

The diversity of the gut microbiota in infant mice was recovered by the sixth week, while relative abundance of *Ruminococcaceae_UCG_014* was low and did not return to normal levels. Gut microbiota in young adult mice recovered in the fourth week. Prevotellaceae and *Alloprevotella* were significantly higher in the high-fat-diet group than those in the control group. The elderly mice had three, two, four, and seven statistically different genera between the dysbiosis and control groups at weeks 6, 8, 10, and 12, respectively. Intestinal epithelial structure and cecum index are restored with microbiota repaired.

**Discussion:**

The gut microbiota in infant and adult mice is more capable of self- resilience, the composition of the microbiota and mucosal morphology of the intestine can be largely restored. Adding protein and fat to the diet accelerated colony recovery in young adult mice in the short term. In elderly mice, the resilience of the gut microbiota was reduced, and the occurrence of dysbiosis at this stage may accelerate organismal aging and affect the lifespan. A limitation of this study is that all data were derived from mice. Therefore, we must be cautious about translating the microbiome results from mice to humans.

## 1 Introduction

The human gastrointestinal tract is the most important symbiotic site for commensal microorganisms. An increasing number of studies have shown that the intestinal microbiome plays an important role in human health and disease (O’Hara and Shanahan, 2006). The total number of bacteria colonizing the gastrointestinal tract is enormous and diverse, with microorganisms weighing approximately one-third of the dry weight of feces, most of which are anaerobic bacteria (Rajilić-Stojanović and de Vos, 2014). Gut microbiome is involved in human metabolism, nutrition, and immunity (Sekirov et al., 2010). The gut bacteria could inhibit pathogen invasion, produce various vitamins, synthesize all essential and non-essential amino acids, and metabolize the non-digestible carbohydrates (Bentley and Meganathan, 1982; Ploux et al., 1992; Pompei et al., 2007; LeBlanc et al., 2013; Magnúsdóttir et al., 2015). The gut bacteria metabolites, such as short-chain fat acids, can also regulate the body’s physiological functions (Morrison and Preston, 2016). Equilibrium among intestinal microorganisms is vital to health, and dysbiosis triggers a series of functional disorders and diseases, such as endocrine and metabolic diseases, autoimmune diseases, neurological degenerative diseases, and the occurrence of tumors (Marchesi et al., 2016).

The composition of the gut microbiome is influenced by diet, age, and the environment and that the current widespread use of antibiotics is one of the most important environmental factors (Wang et al., 2011). It has been reported that antibiotics protect premature babies from potential infections and eliminate the developing intestinal flora (Broadfoot, 2018). For infants and young children on long-term antibiotic-containing medications, the diversity of the gut microbiome is greatly reduced, and drug-resistant microorganisms develop. Although the diversity gradually recovers after the medication is discontinued, early dysbiosis may still cause subsequent health problems, such as increased risk of asthma and obesity and necrotizing small bowel colitis. Additionally, the population is rapidly aging worldwide, and antibiotic-induced gut microbiota dysbiosis is one of the most important factors affecting the health of the older population (Heinsen et al., 2015; Xie et al., 2015).

Given the important role of the gut microbiome in health and disease, it is necessary to understand the development and variations of the gut microbiota. The present study established a rodent model of antibiotic-induced dysbiosis to investigate the self-resilience of the gut microbiome at different ages. In the young group, different diets were added to investigate the effects of dietary factors on microbiome restoration after disruption of the microbiome.

## 2 Materials and methods

### 2.1 Mouse handling and *in situ* sampling

**FIGURE 1:**
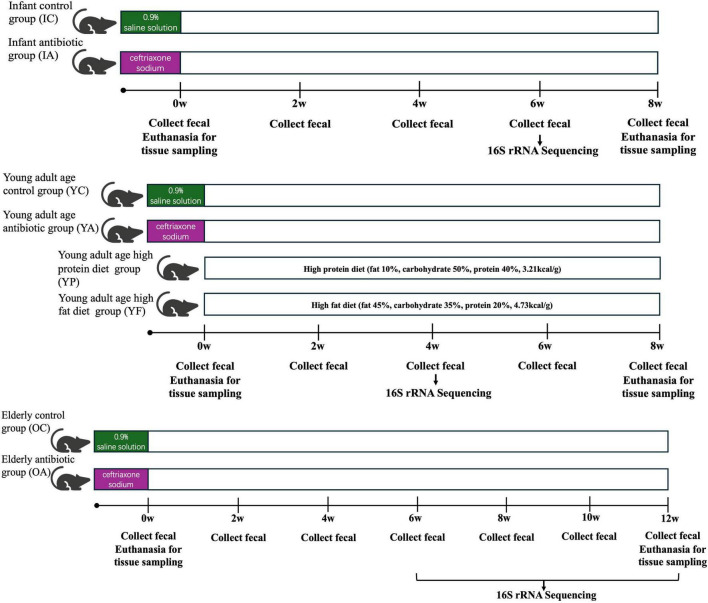
Schematic representation of the animal study.

Sixteen 3-week (infant group), 58 12-week (young adult age group), and 15 72-week (elderly group) female Balb/c mice were selected and reared adaptively in the laboratory for 3 days. Infant mice were randomly divided into the infant control group (IC) (*n* = 8) and the infant antibiotic group (IA) (*n* = 8) groups. The young adult age group mice were randomly divided into a young adult age control group (YC) (*n* = 16) and a young adult age antibiotic group (YA) (*n* = 42). Elderly mice were randomly divided into an elderly control group (OC) (*n* = 5) and an elderly antibiotic group (OA) (*n* = 10). Mice in all the antibiotic groups were orally administered 0.2 mL/day of ceftriaxone sodium at 800 mg/mL dissolved in 0.9% saline solution for 7 days to induce gastrointestinal dysbiosis (Yin et al., 2015). Mice in all control groups were administered 0.2 mL/day of 0.9% saline solution for 7 days in parallel. The YA group was then divided into a young adult high-protein diet group (YP) (*n* = 13) and a young adult high-fat diet (HFD) group (YF) (*n* = 13), which were fed a high-protein (fat 10%, carbohydrate 50%, protein 40%, 3.21kcal/g) and high-fat (fat 45%, carbohydrate 35%, protein 20%, 4.73kcal/g) diet, respectively. Mice were provided with 250 ml of water and 150 g of feed per cage at the beginning of each week, and the remaining water and feed were weighed at the end of each week as a record of the diet. The body weights were recorded weekly and the feces were collected biweekly. Half of all the antibiotic groups were sacrificed at the end of antibiotic administration, and the remaining young adult, infant, and elderly mice were sacrificed at weeks 6, 8, and 12 (Figure 1).

A 0.5-cm ileum segment was sectioned 1 cm above the ileocecal valve, and a 0.5-cm colon segment was sectioned beneath the ileocecal valve. The intact cecum was sectioned and weighed. All the faecalis were frozen at -80 °C. The ileum and colon segment tissues were fixed in a 10% neutral formalin solution and stored at 4 °C.

All the animals were bought from Junke Bioengineering Co., Ltd. (Nanjing, Jiangsu, China) and acclimated in Dalian Medical University Laboratory Animal Center under standard laboratory conditions (ventilated room, 25 ± 1°C, 60 ± 5 % humidity, 12 h light/dark cycle) and had free access to standard water and food (SYXK 2019-0029). The animal study was reviewed and approved by the Biomedical Ethics Committee of Dalian Medical University. All experiments were performed in accordance with the relevant guidelines and regulations of the ethics committee.

### 2.2 Morphology of intestinal segments and structure of the mucosal barrier

A 0.5-cm segment of the ileum and colon tissues was embedded in paraffin, and then, the tissue blocks were sectioned at 4 μm. Sections were stained with hematoxylin and eosin and observed under an optical microscope (DP73; Olympus, Tokyo, Japan). Scores were generated according to the criteria of the modified scale of Bobin-Dubigeon et al. (2001).

### 2.3 DNA Isolation, PCR-DGGE and 16S rRNA Gene Sequencing

An E.Z.N.A. Stool DNA kit (Omega Bio-Tek, Norcross, GA, United States) was used for whole DNA extraction from the stool. The DNA concentration was measured with NanoDrop2000 (Thermo Fisher Scientific, Wilmington, DE, USA).

Use Polymerase Chain Reaction (PCR) with primers GC-338F (5′-CGCCCGGGGCGCGCCCCGG GCGGGGCGGGGGCACGGGGGGCCTACGGGAGGCAGCAG-3′) and 518R (5′-ATTACCGCGGCTGCTGG-3′) amplified the V3 region of the bacterial 16S rRNA gene (Wu et al., 2016). PCR reactions were performed on an ABI GeneAmp^®^ 9700 system (Thermo Fisher Scientific, Wilmington, DE, USA). Denaturing gradient gel electrophoresis (DGGE) was carried out using Molecular Imager Gel Doc™ XR+ (Bio-Rad, Hercules, San Diego, CA, USA), at 60°C and a charge of 80 V for 6.5 h in 7% polyacrylamide gel containing 35 to 65% gradient of denaturants (100% was defined as 40% formamide and 7 M urea). The gel was stained by ethidium bromide for 20 min, then the patterns were visualized by an UV transilluminator. The patterns were digitalised for later analysis by taking a photograph.

For 16S rRNA Gene Sequencing of gut microbiota, PCR was applied to the bulk DNA with the barcoded primers 338F (5′- ACTCCTACGGGAGGCAGCAG-3′) and 806R (5′-GGACTACHVGGGTWTCTAAT-3′), which cover the V3–V4 regions of the 16S rRNA gene (Liu et al., 2016). PCR reactions were performed on an ABI GeneAmp^®^ 9700 system (Thermo Fisher Scientific, Wilmington, DE, USA). The 16S amplicons were purified with a GeneJET PCR Purification Kit (Thermo Fisher Scientific, Waltham, MA, United States). A DNA library was constructed by using the NEXTFLEX Rapid DNA-Seq Kit (Thermo Fisher Scientific, Waltham, MA, United States). The libraries were then sequenced by MiSeq PE300 platform (Illumina, San Diego, CA, United States).

### 2.4 Bioinformatic and statistical analysis

DGGE patterns were analyzed using the Quantity One software (Bio-Rad, Hercules, San Diego, CA, USA). UPGMA method was used for cluster analysis, Calculation of Shannon wiener index and richness from the brightness and number of bands.

Sequences with ≥ 97% similarity were clustered into the same operational taxonomic units (OTUs) by UPARSE (version 11)^1^ (Stackebrandt and Goebel, 1994; Edgar, 2013). The taxonomy of representative sequences for OTUs was determined using RDP Classifier 2.2 (SourceForge, San Diego, CA, USA) against the 16S rRNA gene database using a confidence threshold of 0.7 (Wang et al., 2007). PICRUSt2 was performed using the OmicStudio Analysis^2^ to predict the functional profiles of intestinal microbiome (Douglas et al., 2020). The data were analyzed on the online platform of Majorbio Cloud Platform.^3^ Indices of alpha diversity were calculated based on OTUs using mothur (versionv1.30.2)^4^ (Schloss et al., 2009). *T*-test was used for analysing the differences between two groups, and ANOVA was used for analysing the differences among up to three groups, with *p* < 0.05 being considered a significant difference. The Welch’s *t*-test within STAMP was used to identify phylum, family, and genus that showed significant differences in abundance between groups.

## 3 Results

### 3.1 Basic indicators for mice

**FIGURE 2:**
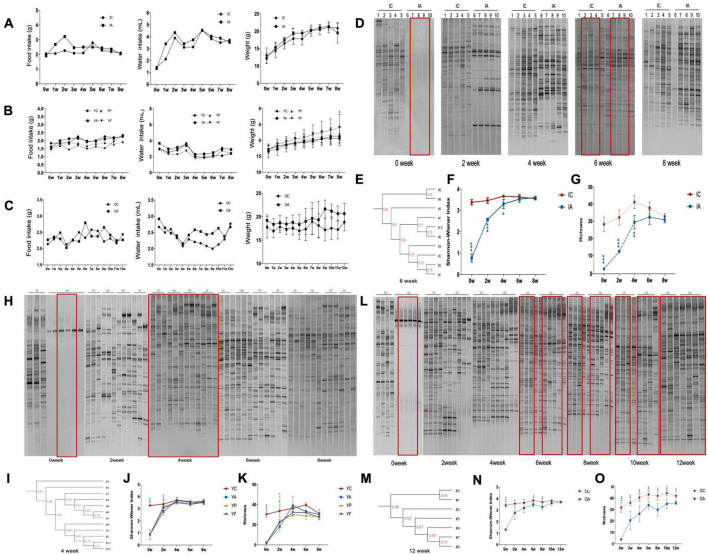
Denaturing gradient gel electrophoresis analysis of gut microbiota in infant, young adult, and elderly mice and the diet, body weight of mice. The diet and the body weight of infant **(A)**, young adult **(B)**, and elderly mice **(C)**; denaturing gradient electrophoresis graph of gut microbiota in infant **(D)**, young adult **(H)**, and elderly mice **(L)**; UPGMA similarity cluster analysis of gut microbiota in infant **(E)**, young adult **(I)**, and elderly mice **(M)**; Shannon-winner index of gut microbiota in infant **(F)**, young adult **(J)**, and elderly mice **(N)**; richness of gut microbiota in infant **(G)**, young adult **(K)** and elderly mice **(O)**. IC, infant control group; IA, infant antibiotic group; YC, young adult age control group; YA, young adult age antibiotic group; YP, young adult high-protein diet group; YF, young adult high-fat diet group; OC, elderly control group; OA, elderly antibiotic group. * Comparing with control group significant difference with *p* < 0.05, ** comparing with control group significant difference with *p* < 0.01, *** comparing with control group significant difference with *p* < 0.001, # comparing with YA group significant difference with *p* < 0.05.

During the experiment, no apparent changes were observed with the hair, activity status and the fecal traits of all mice across different age groups, suggesting minimal stress and an overall healthy disposition of the animals. Food intake, water intake, and body weight of the infant mice increased (Figure 2A). Antibiotic-treated mice had slightly higher food and water intake than the control group. In the first 7 weeks, the weight of the antibiotic-treated mice was higher than that of the control group, with the third week being significant (*p* < 0.05).

For the young adult age mice, food intake increased, while water intake was lower than that at the beginning of the experiment. Body weights were elevated in all groups, with young mice fed an HFD being significantly heavier than the controls in the eighth week (*p* < 0.05) (Figure 2B).

The food intake of elderly mice fluctuated over the course of the experiment, and their water intake tended to decline in the first 4 weeks and recover later, with the antibiotic-treated elderly mice recovering more slowly than the controls (Figure 2C).

### 3.2 Recovery of gut microbiota

The intestinal microbiota diversity in the infant group was greatly reduced after antibiotic administration. Follow-up observations revealed that the number of lane bands belonging to the IA group gradually increased, indicating that the diversity gradually recovered (Figure 2D). Cluster analysis showed that the differences in intestinal microbiota between the two groups gradually decreased (Figure 2E). The Shannon index and abundance in the IA group gradually increased and converged with those in the IC group (Figures 2F, G).

The gut microbiota of antibiotic-treated adult mice recovered gradually (Figure 2H). The recovery degree significantly differed among the YP, YF, and YA groups during the second week. The YP and YF groups showed a significant increase in the diversity and abundance of colony structures, with the diversity index no longer statistically different from that of the control group, and only the abundance was slightly lower (*p* < 0.05). The Shannon index was significantly higher in the YP group than that in the YA group (*p* < 0.05) (Figures 2J, K). The diversity indices of the three groups were not statistically different from those of the control group from week 4 onward. In the clustering diagram, groups YP and YF tended to cluster together, whereas groups YA and YC tended to cluster together (Figure 2I).

The gut microbiota of the elderly mice also tended to recover after antibiotic damage (Figure 2L). However, there were fluctuations in recovery, and at week 12, there was no statistically significant difference in the Shannon index between the two groups (Figure 2N). However, the abundance in the OA group was still significantly lower than that in the control group (Figure 2O). Similarly, the cluster plot showed that after 12 weeks, the colony composition of the mice in the OA group gradually approached that of the mice in the OC group (Figure 2M).

Based on the denaturing gradient gel electrophoresis results, we selected the fourth and sixth weeks as recovery nodes for the adult and infant groups to analyze their gut microbiota using 16s rRNA sequencing. Owing to the fluctuation in the recovery period in the elderly group of mice, we used 16s rRNA sequencing to analyze the gut microbiota in the sixth, eighth, tenth, and twelfth weeks to explore the recovery of the bacterial community in elderly mice.

**FIGURE 3:**
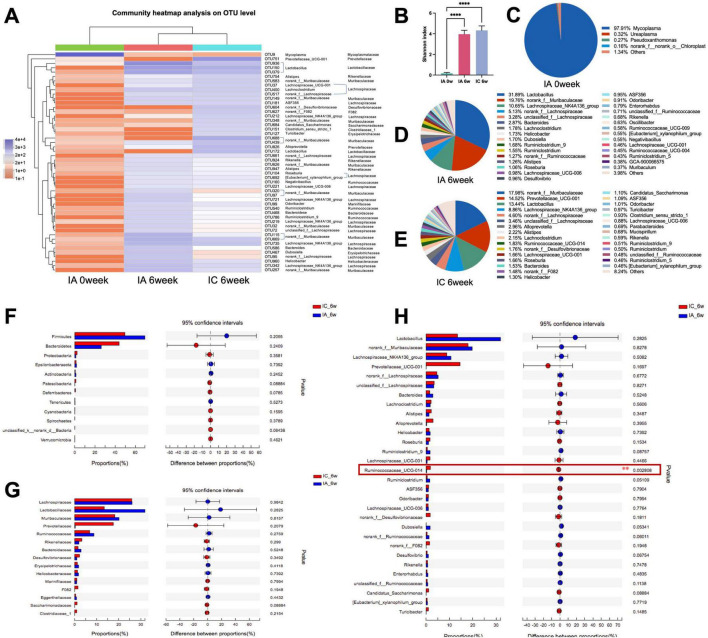
Analysis of the recovery of intestinal microbiome in infant mice. **(A)** Top 50 OTUs heatmap and the taxonomic information; **(B)** Shannon-winner index; **(C–E)** microbiome composition at genus level; analysis of differential microbiome between groups at phylum level **(F)**, family level **(G)**, and genus level **(H)**. IC, infant control group; IA, infant antibiotic group. **p < 0.01, ****p < 0.0001.

The community heat map showed that the community composition at the OUT level of the IA group was significantly different from that after antibiotic infusion (IA 0 week) and gradually converged to that of the IC group at the 6th week (Figure 3A). A comparison of the Shannon indices of the three groups showed that the Shannon index of the IA group was significantly higher than that after antibiotic infusion (*p* < 0.001) and was not statistically different from that of the control group (Figure 3B). This indicates that the alpha diversity of the group IA was restored to a normal level after the 6-week recovery period. Comparing the microbiota composition at the genus level among the three groups, after the instillation of antibiotics, the highest percentage of genus in group IA was *Mycoplasma*, at approximately 97.91% (Figure 3C). After 6 weeks, the microbiota composition of antibiotic-treated infant (IA) mice was significantly changed, with the highest percentage being *Lactobacillus* with approximately 31.89% (Figure 3D), while the *norank_f_Muribaculaceae* (19.76%) and the *Lachnospiraceae_NK4A136_group* (10.65%) were both became the dominant genus. The *norank_f_Muribaculaceae* (17.98%), *Prevotellaceae_UCG-001* (14.52%) and *Lactobacillus* (13.44%) were the dominant genus in control infant (IC) mice (Figure 3E). The between-group comparison showed that the top 13 phylum and the top 15 family were no statistical differences (Figures 3F, G), the top 30 genus were both no statistical differences except for *Ruminococcaceae_UCG_014*, which was slightly lower in the antibiotic-treated infant (IA) mice (Figure 3H). The proportion of coinciding bacteria was 96.67%, which indicated a high degree of recovery.

**FIGURE 4:**
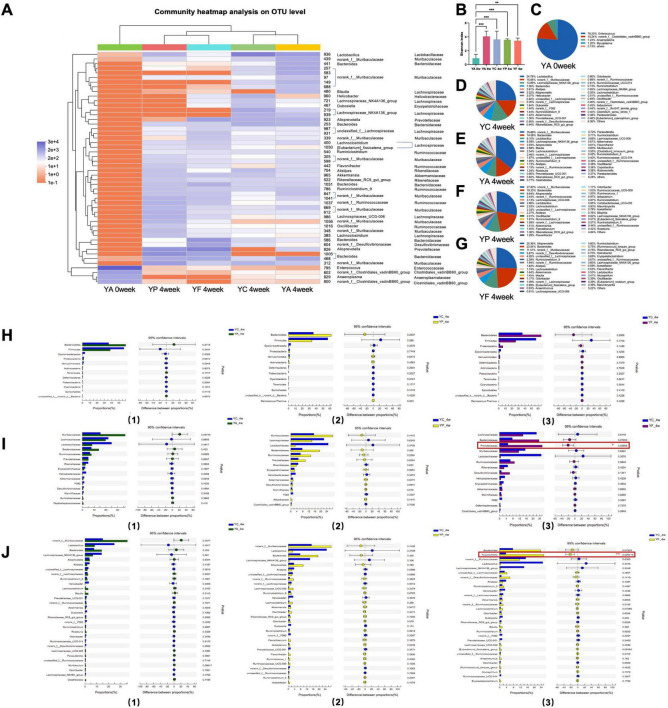
Analysis of the recovery of intestinal microbiome in young adult age mice. **(A)** Top 50 OTUs heatmap and the taxonomic information; **(B)** Shannon-winner index; **(C–G)** microbiome composition at genus level; analysis of differential microbiome between groups at phylum level **(H)**, family level **(I)**, and genus level **(J)**. YC, young adult age control group; YA, young adult age antibiotic group; YP, young adult high-protein diet group; YF, young adult high-fat antibiotic group. *p < 0.05, **p < 0.01, ***p < 0.001.

For young adult age mice, in the 4th week, the community heat map showed that the community composition at the OUT level of the YA, YP, and YF groups was significantly changed (Figure 4A). The Shannon index of these three groups substantially increased, and there was no significant difference from that of the YC group (Figure 4B). Antibiotic treatment altered the genus with the highest percentage of Enterococcus in the gut of adult mice (Figure 4C). After 4 weeks, *norank_f__Muribaculaceae* became the dominant genus in the YA (35.86 %) and YP (27.82 %) groups (Figures 4E, F), *Alloprevotella* (25.36%) became the dominant genu in the YF group (Figure 3G). In YC group (Figure 3D), *Lactobacillus* (24.78%) was the dominant genu. Comparing the YA, YP with the YC group separately, the top 13 phylum, the top 15 family and the top 30 genera have no statistical difference. (Figures 4H, I). However, Prevotellaceae and *Alloprovotella* were significantly more abundant in the YF group than in the control group (Figure 4J).

**FIGURE 5:**
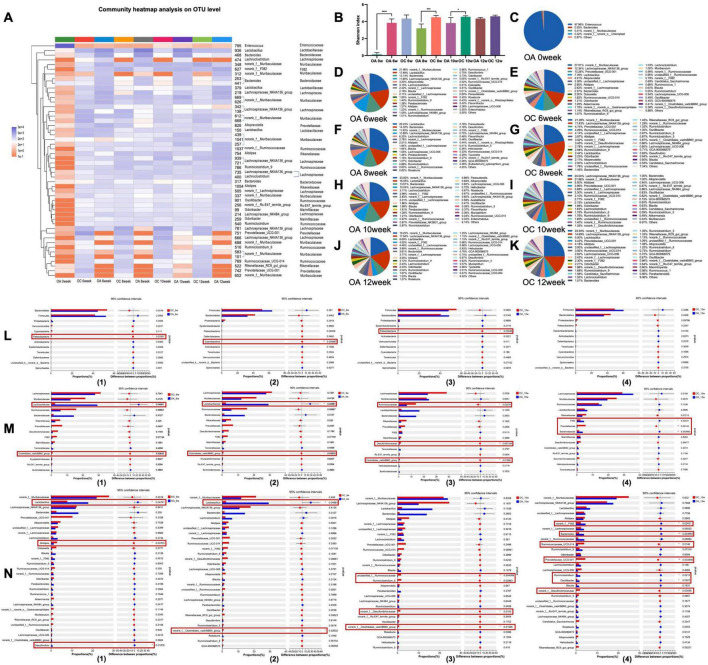
Analysis of the recovery of intestinal microbiome in elderly mice. **(A)** Top 50 OTUs heatmap and the taxonomic information; **(B)** Shannon-winner index; **(C–K)** microbiome composition at genus level; analysis of differential microbiome between groups at phylum level **(L)**, family level **(M)**, and genus level **(N)**. OC, elderly control group; OA, elderly antibiotic group. *p < 0.05, **p < 0.01, ***p < 0.001, ****p < 0.0001.

After 6 weeks of natural recovery, the community heat map showed that the community composition of the elderly mice at the OUT level was significantly different from that after antibiotic administration (OA 0 week) (Figure 5A). The community composition tended to that of the control group, whereas the Shannon index (Figure 5B) was not significantly different from that of the control group. However, recovery was unstable, and the Shannon index of the antibiotic group was significantly lower than that of the control group at 8 and 10 weeks (*p* < 0.001 and *p* < 0.05), respectively) but recovered at 12 weeks. Figures 4C–K demonstrated genus-level microbiota composition at different time points in two group mice, after instilling antibiotics, the dominant genus in group OA was *Enterococcus* (Figure 5C), which became *norank_f__Muribaculaceae* 6 weeks later (Figure 5D). Comparing the OA and OC groups during the same period, at the phylum level (Figure 5L), Patescibacteria in the OA group at the 6 and 10 weeks and Cyanobacteria at the 8 weeks were significantly lower than in the OC group (*p* < 0.05). At the family level (Figure 4M), at 6 and 8 weeks, Lactobacillaceae were significantly higher in the OA group than in the OC group (*p* < 0.05); Clostridiales_vadinBB60_group was significantly lower than that in the OC group (*p* < 0.05). In the 10 weeks, Ruminococcaceae (*p* < 0.05), Desulfovibrionaceae (*p* < 0.01), and Clostridiales_vadinBB60_group (*p* < 0.05) were significantly lower in the OA group than in the OC group. Prevotellaceae in the OA group was significantly lower than that in the OC group in the 12 weeks (*p* < 0.01), while F082 and Bacteroidaceae were significantly higher in the OA group than in the OC group (*p* < 0.05). At the genus level, there were three, two, four, and seven significantly different genera between the two groups at 6, 8, 10, and 12 weeks, respectively (Figure 5N).

### 3.3 Morphology of the ileum and colon and the cecum index

**FIGURE 6:**
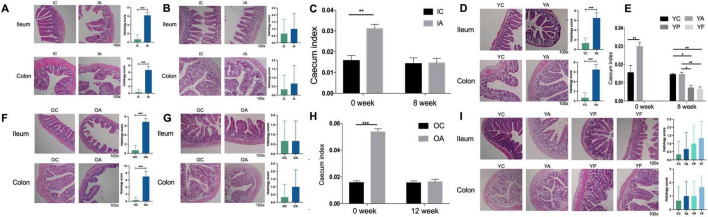
Histological sections with histological scores of ileum and colon, and cecum indices in infant **(C)**, young adult **(E)**, and elderly mice **(H)**. **(A,D,F)** Histological sections of mice with gut microbiome dysbiosis; **(B,G,I)** histological sections of mice at the end of observe. IC, infant control group; IA, infant antibiotic group; YC, young adult age control group; YA, young adult age antibiotic group; YP, young adult high-protein diet group; YF, young adult high-fat antibiotic group; OC, elderly control group; OA, elderly antibiotic group. *p < 0.05, **p < 0.01, ***p < 0.001.

In the antibiotic-treated infant group (Figure 6A), significant swelling and damage were observed in ileal epithelial cells. The villi appeared sparse, disorganized, fragmented and varied significantly in length. The brush border was indistinct, the muscle layer was thin, and the serosa was incomplete. The crypts in the colon were small, shriveled, and structurally incomplete. In contrast, the control group exhibited neatly arranged and dense ileal villi, consistent in length, with a clear brush border, thick muscle layer, and intact serosa. The crypts in the colon were larger and structurally intact. After 8 weeks (Figure 6B), the ileal villi in group IA returned to a neatly arranged state with no breakage, clear brush borders, a thicker muscle layer, and complete serosa. No swelling of the colonic epithelial cells was observed, and the crypts were visible.

In the antibiotic-treated young adult group (Figure 6D), ileal villi were irregularly arranged and fragmented. Ileal epithelial cells exhibited signs of shedding and necrosis. The muscle layer was thinner, the serosa was compromised, and swelling was observed in the colon epithelial cells. In contrast, the control group showed defect-free, neatly and tightly arranged villi and prominent crypts in the colon. After 8 weeks (Figure 6I), the ileal villi in the YA, YP, and YF groups demonstrated an orderly arrangement with no breakage compared to the normal group. The thickness of the muscle layer was restored, and the serosa regained its integrity. No swelling was observed in the colonic epithelial cells, and the crypts were visible. The YA and YP groups exhibited superior recoveries.

In the antibiotic-treated elderly group (Figure 6F), the ileal villi exhibited swelling and fragmentation and were arranged in a disordered manner. The epithelial cells at the apex of the villi showed signs of degeneration and necrosis, and some normal stromal structures were lost. Lymphocyte infiltration was commonly observed in the submucosal layer, and the depth of the colon crypts was reduced, appearing significantly shortened and shriveled. In contrast, the control group exhibited leaf-shaped, neatly arranged, and regularly arranged ileal villi. No signs of necrosis or exfoliation were observed in intestinal epithelial cell layers. The smooth muscle layer was tightly arranged, and the muscle layer was thicker. After 12 weeks (Figure 6G), the ileal villi in the antibiotic-treated elderly group were more neatly arranged than those in the control group. The thickness of the muscle layer was restored, and the serosa appeared more complete. Colon epithelial cells showed no signs of swelling, and the structure of the crypts was more pronounced.

The cecal indices across all groups exhibited similar trends. Following the antibiotic intervention, the cecal indices of the IA, YA, and OA groups were significantly higher than those of the IC, YC, and OC groups (Figures 6C, E, H). After 8 weeks of natural recovery, the cecal index of the IA group normalized (*p* > 0.05). The cecal index of the YA group decreased, with no statistically significant difference from that of the YC group (*p* > 0.05). The cecal indices in the YP (*p* < 0.05) and YF (*p* < 0.01) groups were significantly lower than those in the YA and YC groups. After 12 weeks of natural recovery, the cecal index of the OA group decreased significantly, showing no statistically significant difference from that of the OC group (*p* > 0.05).

### 3.4 Survival analysis and PICRUSt2 analysis of elderly group mice

**FIGURE 7:**
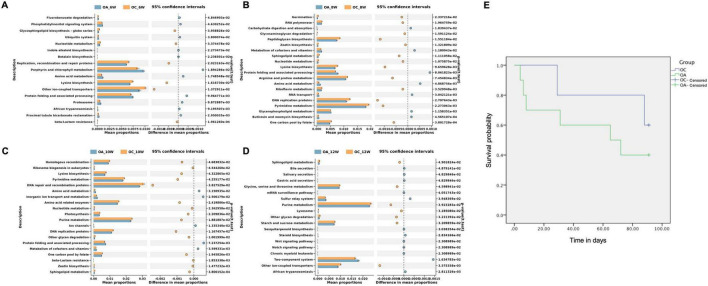
Survival analysis **(E)** of elderly mice and functional profiles of intestinal microbiome. **(A)** Twenty most different functional pathways between OC and OA in week 6 **(A)**, 8 **(B)**,10 **(C)**, and 12 **(D)**. OC, elderly control group; OA, elderly antibiotic group.

PICRUSt2 analysis was used to predict the metagenomic functions associated with bacterial communities based on 16S rRNA sequencing data from the gut microbiota of older mice. At Kyoto Encyclopedia of Genes and Genomes level 3, 17, 37, 20, and 19 different functional pathways were found in the sixth, eighth, tenth, and twelfth weeks, respectively (Figures 7A–D). The lysine biosynthesis pathway was enriched in the control group at the sixth, eighth, and tenth weeks. In the twelfth week, the glycine, serine, and threonine metabolism and sulfur relay system pathways were enriched in the control group. Some older mice died during the experimental observation. The experimental observation period for elder mice totaled 91 days, with four survivors in the experimental group and three in the control group. The survival rate in the OA group was consistently lower than that in the OC group; however, the difference was not statistically significant (*p* = 0.384). However, it has been suggested that failure to restore the intestinal flora of the elderly to baseline levels will result in lower survival and shorter lifespans (Figure 7E).

## 4 Discussion

Tens of thousands of microorganisms colonize the human intestinal tract and are important for maintaining the host’s physiological health (Sonnenburg and Bäckhed, 2016). However, the dynamic balance of intestinal microorganisms can be easily disturbed by various external factors such as antibiotics, diet, and hygiene conditions, leading to disorders, resulting in many dysfunctions and diseases (Round and Mazmanian, 2009; Li et al., 2010; Ridaura et al., 2013; McElhanon et al., 2014; Forslund et al., 2015; Tan et al., 2015; Garcia-Rios et al., 2017). Antibiotics can significantly alter the composition of gut microbiota in the human body(Neuman et al., 2018). Rosa et al. (2018) summarized the effects of various antibiotics on the murine gastrointestinal tract and their systemic repercussions, Ceftriaxone was found to cause minor systemic repercussions except dysbiosis. Ceftriaxone is a third-generation cephalosporin with a wide spectrum of activity against gram-negative bacilli and most gram-positive bacteria. The present study investigated the alterations in the flora of mice of different ages after treatment with antibiotics and intended to gain insight into the ability of the gut microbiota to recover in mice of different ages and the effects of gut microbiota disorder and repair on the host. With reference to several studies, we selected 3-week mice roughly equivalent to 6 months of age in humans as infant, 12-week mice roughly equivalent to 20 years of age in humans as young adult, and 72-week mice roughly equivalent to 56 years of age in humans as elderly (Dutta and Sengupta, 2016; Bilkei-Gorzo et al., 2017; Smithey et al., 2018).

The period from the beginning of the fetal period to the second year of life is considered the early years of life and is the most critical stage in determining the life and health of an organism. Fouhy et al. (2012) used high-throughput sequencing to analyze nine infants who received a combination of ampicillin and gentamicin 48 h after birth and whose gut microbial composition remained incompletely restored after the 8th week. Fouhy et al. (2012) used high-throughput sequencing to analyze nine infants who received a combination of ampicillin and gentamicin 48 h after birth and whose gut microbial composition remained incompletely restored after the 8th week. In the present study, 3-week-old mice were selected to investigate the recovery of the gut microbiota after disruption in the early stages of life. We performed 16s sequencing on the flora of mice that had recovered naturally for 6 weeks to determine the specific recovery situation. The results showed that after antibiotic intervention, the alpha diversity of the intestinal flora of the infant group decreased, the number of Firmicutes and Bacteroidetes decreased drastically, and Tenericutes occupied the main position. The diversity of the mouse flora was recovered when the natural recovery reached the sixth week. The comparison of the differential flora of different taxonomic levels showed that only the level of Ruminococcaceae UCG 014 in the group IA flora was lower, indicating that the intestinal tract of infant mice still has a strong ability to repair after short-term intervention with high doses of antibiotics.

When an organism enters adulthood, the organs and systems are mature and fully functional, metabolism is vigorous, immunity is high, and it is the stage in which the three age groups have the strongest ability to resist external interference. Many studies have shown that bacteria in the gut of adult organisms are highly resilient to damage (Dethlefsen and Relman, 2011). Palleja et al. (2018) found that after administering three antibiotics, flora recovery occurred in healthy adult subjects. The present study selected Balb/c mice at 3 months of age to reflect the recovery of intestinal flora in adulthood. The intestinal flora of adult mice is highly reparative and can be restored in only 4 weeks. The diversity of the intestinal flora almost returned to normal by the fourth week. Further analyses using 16S rDNA sequencing similarly confirmed our view that there were no statistically significant differences between the YA and control groups at the phylum, family, and genus levels.

The gut flora also tended to recover in elderly mice after cessation of oral antibiotics. The present study found no differences between the experimental and control flora at the phylum level in elderly mice at the twelfth week after cessation of antibiotic administration. However, at the genus level, seven genera showed significant differences between the two groups in the twelfth week. Only three, two, and four genera showed significant differences in the sixth, eighth, and tenth weeks, respectively.

Metabolites produced by dietary nutrition are important bridges between the gut microbiota and an organism’s immunity and nutrition and play an important role in regulating organismal homeostasis. Certain microorganisms in the gut are sensitive to the proportions of dietary components and behave differently under different temporal and geographic conditions concerning body nutrition (Turnbaugh et al., 2009). Dietary factors alter the abundance of flora and affect growth kinetics (Korem et al., 2015). Considering that the body functions in the infant and elderly stages are of relative immature or aged, only adult mice were selected in exploring the effects of high protein and high fat diets on changes in gut bacterial composition in the presence of dysbiosis. In healthy organisms, the abundance of Firmicutes and Bacteroidetes accounts for approximately 98% of the intestinal tract. Firmicutes promote the absorption of dietary energy, whereas Bacteroidetes play an important role in the metabolism of polysaccharides and steroids, which together maintain the energy metabolism of the organism and the stability of the physiological functions of the intestinal tract (Haller and Hörmannsperger, 2015; Tabibian et al., 2016). In the present study, we found that the ratio of Firmicutes to Bacteroidetes was significantly altered after antibiotic intervention, with a significant increase in Firmicutes and a significant decrease in Bacteroidetes. Denaturing gradient gel electrophoresis showed that high-fat and high-protein diets accelerated flora recovery in antibiotic-treated mice and that the diversity of flora in the high-protein diet group was significantly higher than that in the naturally recovering group in the second week. Analysis of the results from the fourth week using 16S rRNA gene sequencing showed that the YA and YP groups recovered to a high degree, with the intestinal flora reaching normal levels. The abundance of Prevotellaceae and *Alloprevotella* in the YF group remained elevated.

Gut microbiota dysbiosis is often accompanied by alterations in intestinal permeability. We found that mucosal morphology changed in the ileum and colon of all mice treated with antibiotics. After the flora recovered, the mucosal morphology also recovered. It has been shown that *Lactobacillus* and *Bacteroides* can metabolize exogenous plant fibers to produce short-chain fat acids, which are absorbed by the epithelial cells and can, in turn, contribute to their proliferation and development (Wang et al., 2017). Potential pathogenic microorganisms such as *Enterococcus* in the body pose a risk of inducing enteritis (Hong et al., 2011; Strickertsson et al., 2013). In the present study, we found that *Lactobacillus* and *Bacteroides* were slightly higher in infant and elderly mice whose colonies were restored after antibiotic treatment than in the control group. *Bacteroides* were slightly higher in the young adult group than in the control group, which may be related to the repair of mucosal damage. In this study, mucosal morphology recovered better in the young adult experimental group fed a high-protein diet. Beaumont et al. (2017) found that a 2-week period of high-protein dietary intake modulated the expression of genes involved in intestinal cell metabolism and proliferation, thereby reducing the development of intestinal mucosal inflammation. A previous study by Bansal et al. (2010) also suggested that when consuming large amounts of proteins, organisms can generate high concentrations of indole substances via microbial metabolism, leading to an increase in tight junctions between epithelial cells and a decrease in the production of inflammatory factors. Protein supplementation promotes the recovery of intestinal mucosal morphology during intestinal microbiota dysbiosis.

Recent studies have shown that the composition of intestinal microbiota is related to the aging and lifespan of an organism (Parker et al., 2022). Antibiotic-treated mice in this study had a shorter lifespan. In view of the poorer recovery of the gut microbiota and shorter lifespan in elderly mice, we proceeded to analyze gut microbiota function across this age group. Functional prediction revealed that glycine, serine, and threonine metabolism were significantly lower in elderly experimental mice than in control mice. Aon et al. (2020) found that glycine, serine, and threonine metabolism levels were positively correlated with lifespan in mice, which may play a role in resisting organismal aging. The sulfur relay system pathway is enriched in patients with Crohn’s disease (Metwaly et al., 2020), and this study revealed its enrichment in antibiotic-treated elderly mice. This suggests that a disturbed state of gut microbiota still exists in antibiotic-treated elderly mice.

The present study examined the ability of the gut microbiota to recover from antibiotic-induced dysbiosis in mice of different ages. In summary (Table 1), the intestinal flora in infant and adult mice is more capable of self-healing, and its composition and mucosal morphology can be largely restored. The recovery period was 6 weeks for infant mice and 4 weeks for adult mice. Adding protein and fat to the diet accelerated colony recovery in middle-aged mice in the short term. In older mice, the resilience of the gut flora is reduced, and the occurrence of flora disorders at this stage may accelerate organismal aging and affect the lifespan. However, caution should be exercised when translating mouse microbiome profiles to humans. Further research should be conducted to examine whether antibiotic use in the elderly should be followed by intervention for the flora disorders it causes to promote a return to homeostasis.

**TABLE 1:** Recovery of gut microbiota in mice of all ages.

Group	Phylum (%)	Famliy (%)	Genus (%)
IA	100	100	96.67
YA	100	100	100
YP	100	100	100
YF	100	93.33	96.67
OA	100	80	76.67

## Data Availability

Raw sequencing data are available in the NCBI Sequence Read Archive (https://www.ncbi.nlm.nih.gov/sra) under study accession PRJNA1096257.

## Footnotes

1
http://drive5.com/uparse/

2
https://www.omicstudio.cn/analysis/

3
www.majorbio.com

4
http://www.mothur.org/wiki/Calculators
